# 3β-Chloro-5α-cholestan-6-one

**DOI:** 10.1107/S1600536812012482

**Published:** 2012-03-28

**Authors:** Samina Khan Yusufzai, Hasnah Osman, Aisyah Saad Abdul Rahim, Suhana Arshad, Ibrahim Abdul Razak

**Affiliations:** a School of Chemical Sciences, Universiti Sains Malaysia, 11800 USM, Penang, Malaysia; b School of Pharmaceutical Sciences, Universiti Sains Malaysia, 11800 USM, Penang, Malaysia; c School of Physics, Universiti Sains Malaysia, 11800 USM, Penang, Malaysia

## Abstract

The asymmetric unit of the title compound, C_27_H_45_ClO, consists of two crystallographically independent mol­ecules. In both mol­ecules, the three cyclo­hexane rings in the steroid fused-ring systems adopt chair conformations, while the cyclo­pentane ring adopts a half-chair conformation in one mol­ecule and an envelope conformation in the other. In the crystal, the mol­ecules are linked into a two-dimensional network by weak C—H⋯O hydrogen bonds. The crystal studied is a nonmerohedral twin with a refined ratio of twin components of 0.264 (3):0.736 (3).

## Related literature

For a crystallographic comparison of cholesterols, see: Rajnikant *et al.* (2006 ▶). For the biological activity of steroidal derivatives, see: Pluempe & Pulls (1971 ▶); Sawhney *et al.* (1975 ▶); Yonova & Stoilkova (2004 ▶). For related structures, see: Yusufzai *et al.* (2012 ▶); Ketuly *et al.* (2011 ▶). For ring conformations, see: Cremer & Pople (1975 ▶). For the synthesis, see: Windaus & Dalmer (1919 ▶). For the stability of the temperature controller used for data collection, see: Cosier & Glazer (1986 ▶).
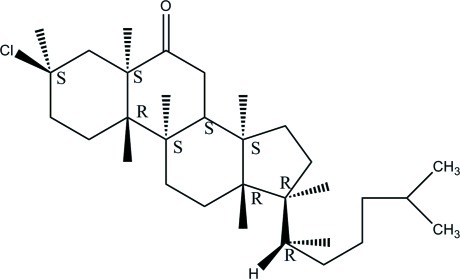


## Experimental

### 

#### Crystal data

C_27_H_45_ClO
*M* *_r_* = 421.08
Monoclinic, 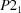

*a* = 7.6603 (3) Å
*b* = 15.7249 (6) Å
*c* = 20.8434 (8) Å
β = 94.069 (2)°
*V* = 2504.41 (17) Å^3^
*Z* = 4
Mo *K*α radiation
μ = 0.17 mm^−1^
*T* = 100 K
0.25 × 0.18 × 0.14 mm

#### Data collection

Bruker SMART APEXII CCD area-detector diffractometer
Absorption correction: multi-scan (*SADABS*; Bruker, 2009 ▶) *T*_min_ = 0.960, *T*_max_ = 0.977
14186 measured reflections
14186 independent reflections
10360 reflections with *I* > 2σ(*I*)
*R*_int_ = 0.000

#### Refinement

*R*[*F* ^2^ > 2σ(*F* ^2^)] = 0.067
*wR*(*F* ^2^) = 0.179
*S* = 1.02
14186 reflections
534 parameters
1 restraint
H-atom parameters constrained
Δρ_max_ = 0.74 e Å^−3^
Δρ_min_ = −0.34 e Å^−3^
Absolute structure: Flack (1983 ▶), 6535 Friedel pairs
Flack parameter: 0.03 (6)

### 

Data collection: *APEX2* (Bruker, 2009 ▶); cell refinement: *SAINT* (Bruker, 2009 ▶); data reduction: *SAINT*; program(s) used to solve structure: *SHELXTL* (Sheldrick, 2008 ▶); program(s) used to refine structure: *SHELXTL*; molecular graphics: *SHELXTL*; software used to prepare material for publication: *SHELXTL* and *PLATON* (Spek, 2009 ▶).

## Supplementary Material

Crystal structure: contains datablock(s) global, I. DOI: 10.1107/S1600536812012482/lh5434sup1.cif

Structure factors: contains datablock(s) I. DOI: 10.1107/S1600536812012482/lh5434Isup2.hkl

Additional supplementary materials:  crystallographic information; 3D view; checkCIF report

## Figures and Tables

**Table 1:** Hydrogen-bond geometry (Å, °)

*D*—H⋯*A*	*D*—H	H⋯*A*	*D*⋯*A*	*D*—H⋯*A*
C7*A*—H7*AA*⋯O1*A*^i^	0.99	2.37	3.349 (4)	168
C7*B*—H7*BA*⋯O1*B*^i^	0.99	2.47	3.265 (4)	137
C23*A*—H23*B*⋯O1*B*^ii^	0.99	2.51	3.414 (4)	152
C23*B*—H23*D*⋯O1*A*^iii^	0.99	2.50	3.453 (4)	161

Symmetry codes: (i) 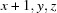; (ii) 
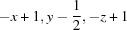
; (iii) 
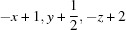
.

**Fig. 1.**
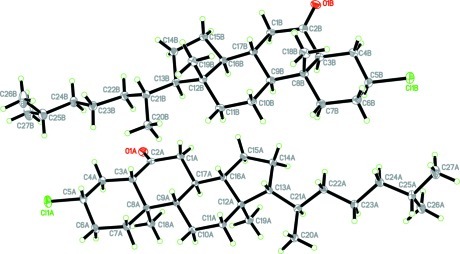
The molecular structure of the title compound, showing 30% probability displacement ellipsoids.

**Fig. 2.**
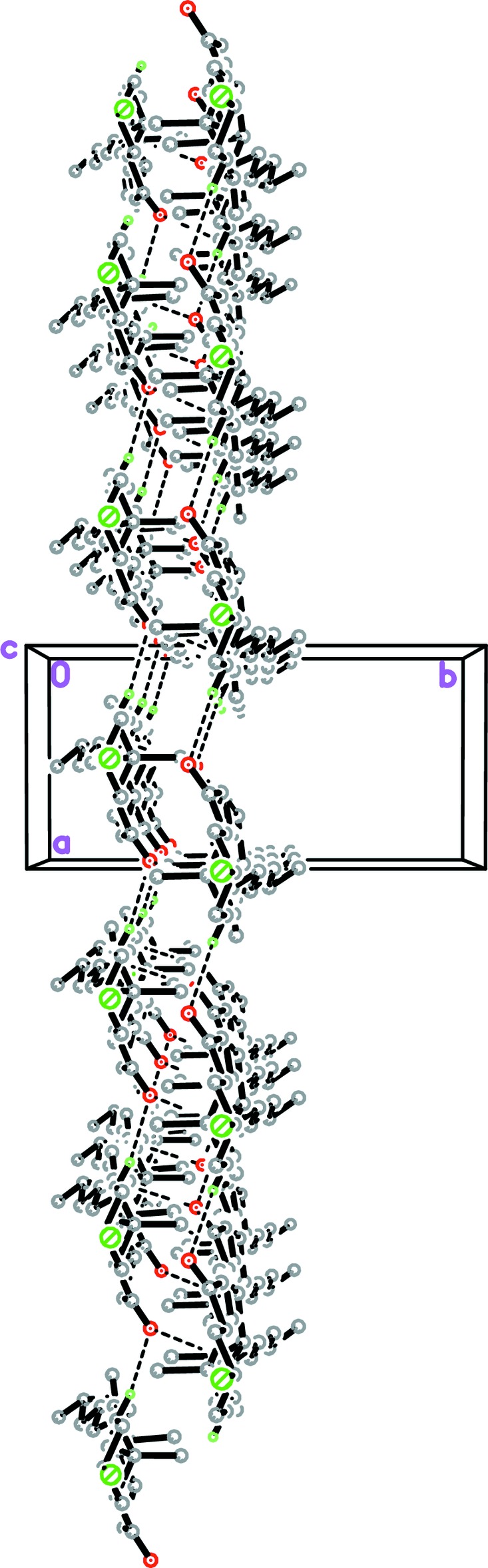
The crystal packing of the title compound. For clarity, those H atoms not involved in the intermolecular interactions (dashed lines) have been omitted.

## supplementary crystallographic information

### Comment

The cholesterol molecule in steroidal chemistry is well known as it comprises of a four-ring structure of which three are six-membered cyclohexane rings and one is a five membered carbon ring (Rajnikant *et al.*, 2006). The importance of steroidal compounds has long been recognized in the field of synthetic organic chemistry and steroidal derivatives are an important group of natural compounds possessing a variety of biological activities such as antidiabetic, bactericidal, fungicidal, herbicidal and algeacidal activities (Pluempe & Pulls, 1971; Sawhney *et al.*, 1975; Yonova & Stoilkova, 2004). In the present work an attempt has been made to synthesize a derivative of the cholesterol molecule. The crystal structure of the title compound is presented herein.

The asymmetric unit of the title compound (Fig. 1), consists of two crystallographically independent molecules *A* and *B*. The bond lengths and angles are within normal ranges and are comparable to the related structures (Yusufzai *et al.*, 2012; Ketuly *et al.*, 2011). In molecule *A*, the cyclohexane (C1A–C3A/C8A/C9A/C17A, C3A–C8A & C9A–C12A/C16A/C17A) rings adopt chair conformation (Cremer & Pople, 1975) [puckering parameters Q= 0.560 (3) Å, Θ= 169.0 (3)° and Φ= 353.7 (15)°; Q= 0.571 (3) Å, Θ= 180.0 (3)° and Φ= 283.0 (17)° and Q= 0.577 (3) Å, Θ= 173.9 (3)° and Φ= 54.0 (3)°, respectively] while the cyclopentane (C12A–C16A) ring is twisted about the C16A—C12A bond [puckering parameters Q= 0.452 (3) Å and φ= 349.1 (4)°], thereby adopting a half-chair conformation. Meanwhile, in molecule *B*, the cyclohexane (C1B–C3B/C8B/C9B/C17B, C3B–C8B & C9B–C12B/C16B/C17B) rings adopt chair conformations [puckering parameters Q= 0.562 (3) Å, Θ= 167.4 (3)° and Φ= 352.5 (14)°; Q= 0.564 (3) Å, Θ= 178.4 (3)° and Φ= 298.0 (8)° and Q= 0.569 (3) Å, Θ= 175.7 (3)° and Φ= 52.0 (4)°, respectively] and the cyclopentane (C12B–C16B) ring is in envelope conformation with puckering parameters Q= 0.456 (3) Å and φ= 351.4 (4)° with atom C12B at the flap.

There are nine chiral centres presented in each molecule. From the structure presented, these centers exhibit the following relative chiralities: C3A/C3B = *S*; C5A/C5B = *S*; C8A/C8B = *R*; C9A/C9B = *S*; C12A/C12B = *R*; C13A/C13B = *R*; C16A/C16B = *S*; C17A/C17B = *S* and C21A/C21B = *R*.

The crystal packing is shown in Fig. 2. The molecules are connected by C7A—H7AA···O1A^i^, C7B—H7BA···O1B^i^6, C23A—H23B···O1B^ii^ and C23B—H23D···O1A^iii^ intermolecular hydrogen bonds (Table 1) into a two-dimensional network parallel to *ac* plane.

### Experimental

A solution of 3β-chloro-6-nitrocholest-5-ene (12 g) and glacial acetic acid (240 ml) was heated just to get a clear solution. Then zinc dust (24 g) was added gradually in small portions with constant shaking. The suspension was heated under reflux for 4 h and water (24 ml) was added at regular intervals during the course of reaction. The hot solution was poured into ice-cold water. The organic matter was extracted with ether and ethereal solution was washed successively with water, sodium bicarbonate solution (5%) and again with water and dried over anhydrous sodium sulfate. Evaporation of the solvent furnished the ketone as an oil which was crystallized from methanol to give shiny needle like crystals, *m.p.*: 401-402K [reported *m.p.* 402K (Windaus & Dalmer, 1919)].

### Refinement

All H atoms were positioned geometrically [C–H = 0.98–1.0 Å] and refined using a riding model with *U*_iso_(H) = 1.2 or 1.5 *U*_eq_(C). A rotating group model was applied to the methyl groups. 6535 Friedel pairs were used to determine the absolute configuration. The crystal studied is a non-merohedral twin with the refined ratio of twin components of 0.264 (3): 0.736 (3).

### Crystal data

**Table:**

C_27_H_45_ClO	*F*(000) = 928
*M**_r_* = 421.08	*D*_x_ = 1.117 Mg m^−^^3^
Monoclinic, *P*2_1_	Mo *K*α radiation, λ = 0.71073 Å
Hall symbol: P 2yb	Cell parameters from 5836 reflections
*a* = 7.6603 (3) Å	θ = 2.8–30.1°
*b* = 15.7249 (6) Å	µ = 0.17 mm^−^^1^
*c* = 20.8434 (8) Å	*T* = 100 K
β = 94.069 (2)°	Block, colourless
*V* = 2504.41 (17) Å^3^	0.25 × 0.18 × 0.14 mm
*Z* = 4	

### Data collection

**Table:**

Bruker SMART APEXII CCD area-detector diffractometer	14186 independent reflections
Radiation source: fine-focus sealed tube	10360 reflections with *I* > 2σ(*I*)
Graphite monochromator	*R*_int_ = 0.000
φ and ω scans	θ_max_ = 30.2°, θ_min_ = 1.0°
Absorption correction: multi-scan (*SADABS*; Bruker, 2009)	*h* = −10→10
*T*_min_ = 0.960, *T*_max_ = 0.977	*k* = −22→22
14186 measured reflections	*l* = −21→29

### Refinement

**Table:**

Refinement on *F*^2^	Secondary atom site location: difference Fourier map
Least-squares matrix: full	Hydrogen site location: inferred from neighbouring sites
*R*[*F*^2^ > 2σ(*F*^2^)] = 0.067	H-atom parameters constrained
*wR*(*F*^2^) = 0.179	*w* = 1/[σ^2^(*F*_o_^2^) + (0.0924*P*)^2^ + 0.2267*P*] where *P* = (*F*_o_^2^ + 2*F*_c_^2^)/3
*S* = 1.02	(Δ/σ)_max_ = 0.001
14186 reflections	Δρ_max_ = 0.74 e Å^−^^3^
534 parameters	Δρ_min_ = −0.34 e Å^−^^3^
1 restraint	Absolute structure: Flack (1983), 6535 Friedel pairs
Primary atom site location: structure-invariant direct methods	Flack parameter: 0.03 (6)

### Special details

**Table:**

Experimental. The crystal was placed in the cold stream of an Oxford Cryosystems Cobra open-flow nitrogen cryostat (Cosier & Glazer, 1986) operating at 100.0 (1) K.
Geometry. All e.s.d.'s (except the e.s.d. in the dihedral angle between two l.s. planes) are estimated using the full covariance matrix. The cell e.s.d.'s are taken into account individually in the estimation of e.s.d.'s in distances, angles and torsion angles; correlations between e.s.d.'s in cell parameters are only used when they are defined by crystal symmetry. An approximate (isotropic) treatment of cell e.s.d.'s is used for estimating e.s.d.'s involving l.s. planes.
Refinement. Refinement of *F*^2^ against ALL reflections. The weighted *R*-factor *wR* and goodness of fit *S* are based on *F*^2^, conventional *R*-factors *R* are based on *F*, with *F* set to zero for negative *F*^2^. The threshold expression of *F*^2^ > σ(*F*^2^) is used only for calculating *R*-factors(gt) *etc*. and is not relevant to the choice of reflections for refinement. *R*-factors based on *F*^2^ are statistically about twice as large as those based on *F*, and *R*- factors based on ALL data will be even larger.

### Fractional atomic coordinates and isotropic or equivalent isotropic displacement parameters (Å^2^)

**Table:**

	*x*	*y*	*z*	*U*_iso_*/*U*_eq_	
Cl1A	0.95587 (11)	0.43447 (6)	1.15732 (3)	0.0373 (2)	
O1A	0.5385 (3)	0.36817 (15)	0.94654 (11)	0.0309 (5)	
C1A	0.6511 (3)	0.43069 (19)	0.85333 (13)	0.0217 (5)	
H1AA	0.6273	0.4923	0.8481	0.026*	
H1AB	0.5537	0.3995	0.8302	0.026*	
C2A	0.6558 (3)	0.40841 (18)	0.92389 (13)	0.0213 (5)	
C3A	0.8149 (3)	0.43897 (19)	0.96392 (13)	0.0207 (5)	
H3AA	0.8174	0.5022	0.9593	0.025*	
C4A	0.8036 (4)	0.4207 (2)	1.03561 (13)	0.0246 (6)	
H4AA	0.6965	0.4470	1.0508	0.030*	
H4AB	0.7975	0.3586	1.0428	0.030*	
C5A	0.9647 (4)	0.4573 (2)	1.07281 (13)	0.0257 (6)	
H5AA	0.9636	0.5204	1.0671	0.031*	
C6A	1.1334 (4)	0.4230 (2)	1.04917 (13)	0.0281 (7)	
H6AA	1.1404	0.3609	1.0570	0.034*	
H6AB	1.2344	0.4502	1.0733	0.034*	
C7A	1.1416 (4)	0.4408 (2)	0.97677 (13)	0.0268 (6)	
H7AA	1.2504	0.4157	0.9621	0.032*	
H7AB	1.1471	0.5030	0.9700	0.032*	
C8A	0.9838 (3)	0.40478 (18)	0.93530 (13)	0.0194 (6)	
C9A	0.9845 (3)	0.43831 (19)	0.86532 (12)	0.0194 (5)	
H9AA	0.9757	0.5016	0.8683	0.023*	
C10A	1.1523 (4)	0.4202 (2)	0.83238 (13)	0.0242 (6)	
H10A	1.1709	0.3579	0.8314	0.029*	
H10B	1.2522	0.4456	0.8584	0.029*	
C11A	1.1515 (3)	0.4548 (2)	0.76347 (13)	0.0226 (6)	
H11A	1.1527	0.5177	0.7648	0.027*	
H11B	1.2591	0.4358	0.7441	0.027*	
C12A	0.9914 (3)	0.42491 (18)	0.72111 (13)	0.0185 (5)	
C13A	0.9501 (4)	0.47380 (18)	0.65620 (13)	0.0194 (5)	
H13A	0.9715	0.5355	0.6652	0.023*	
C14A	0.7497 (4)	0.46238 (19)	0.64270 (13)	0.0231 (6)	
H14A	0.7248	0.4171	0.6103	0.028*	
H14B	0.6959	0.5160	0.6260	0.028*	
C15A	0.6741 (3)	0.4377 (2)	0.70748 (12)	0.0225 (5)	
H15A	0.5754	0.4753	0.7169	0.027*	
H15B	0.6334	0.3779	0.7068	0.027*	
C16A	0.8290 (3)	0.45001 (17)	0.75665 (12)	0.0183 (5)	
H16A	0.8384	0.5127	0.7643	0.022*	
C17A	0.8228 (3)	0.40925 (17)	0.82266 (12)	0.0174 (5)	
H17A	0.8293	0.3461	0.8174	0.021*	
C18A	0.9882 (4)	0.3067 (2)	0.93698 (15)	0.0277 (6)	
H18A	1.0948	0.2865	0.9186	0.042*	
H18B	0.8855	0.2843	0.9118	0.042*	
H18C	0.9871	0.2872	0.9816	0.042*	
C19A	0.9991 (4)	0.32876 (18)	0.70822 (15)	0.0244 (6)	
H19A	1.0050	0.2980	0.7492	0.037*	
H19B	1.1031	0.3157	0.6853	0.037*	
H19C	0.8940	0.3113	0.6820	0.037*	
C20A	1.2515 (4)	0.45940 (19)	0.61080 (13)	0.0240 (6)	
H20A	1.3092	0.4518	0.5707	0.036*	
H20B	1.2935	0.4160	0.6419	0.036*	
H20C	1.2788	0.5160	0.6284	0.036*	
C21A	1.0524 (4)	0.45052 (18)	0.59722 (13)	0.0217 (5)	
H21A	1.0266	0.3897	0.5862	0.026*	
C22A	0.9858 (4)	0.50540 (19)	0.53937 (13)	0.0229 (6)	
H22A	0.8582	0.4963	0.5316	0.027*	
H22B	1.0040	0.5660	0.5509	0.027*	
C23A	1.0721 (4)	0.48833 (19)	0.47650 (13)	0.0242 (6)	
H23A	1.1965	0.5059	0.4815	0.029*	
H23B	1.0684	0.4266	0.4672	0.029*	
C24A	0.9802 (4)	0.53656 (19)	0.41997 (15)	0.0261 (6)	
H24A	0.8575	0.5166	0.4143	0.031*	
H24B	0.9776	0.5977	0.4311	0.031*	
C25A	1.0634 (4)	0.5272 (2)	0.35591 (14)	0.0281 (6)	
H25A	1.1907	0.5411	0.3632	0.034*	
C26A	1.0478 (5)	0.4370 (2)	0.33031 (15)	0.0381 (8)	
H26A	1.0945	0.3972	0.3634	0.057*	
H26B	1.1142	0.4317	0.2920	0.057*	
H26C	0.9244	0.4238	0.3190	0.057*	
C27A	0.9819 (6)	0.5897 (2)	0.30635 (17)	0.0430 (9)	
H27A	1.0020	0.6480	0.3217	0.064*	
H27B	0.8557	0.5791	0.3001	0.064*	
H27C	1.0355	0.5821	0.2654	0.064*	
Cl1B	0.48971 (13)	0.70424 (7)	0.33925 (4)	0.0439 (2)	
O1B	0.0567 (3)	0.78060 (15)	0.52100 (10)	0.0317 (5)	
C1B	0.1591 (3)	0.72188 (18)	0.62247 (13)	0.0208 (5)	
H1BA	0.1333	0.6606	0.6275	0.025*	
H1BB	0.0609	0.7544	0.6388	0.025*	
C2B	0.1684 (4)	0.74109 (18)	0.55219 (14)	0.0221 (6)	
C3B	0.3314 (3)	0.7090 (2)	0.52305 (13)	0.0222 (5)	
H3BA	0.3339	0.6460	0.5297	0.027*	
C4B	0.3277 (4)	0.7235 (2)	0.45059 (14)	0.0268 (6)	
H4BA	0.2229	0.6959	0.4292	0.032*	
H4BB	0.3211	0.7852	0.4413	0.032*	
C5B	0.4905 (4)	0.6868 (2)	0.42489 (14)	0.0308 (7)	
H5BA	0.4909	0.6241	0.4328	0.037*	
C6B	0.6570 (4)	0.7241 (2)	0.45813 (15)	0.0347 (8)	
H6BA	0.6627	0.7858	0.4489	0.042*	
H6BB	0.7606	0.6966	0.4413	0.042*	
C7B	0.6589 (4)	0.7099 (2)	0.53135 (14)	0.0290 (7)	
H7BA	0.7643	0.7376	0.5523	0.035*	
H7BB	0.6680	0.6482	0.5402	0.035*	
C8B	0.4956 (4)	0.74503 (19)	0.56156 (14)	0.0221 (6)	
C9B	0.4919 (3)	0.71337 (19)	0.63200 (13)	0.0216 (6)	
H9BA	0.4821	0.6500	0.6293	0.026*	
C10B	0.6575 (3)	0.7313 (2)	0.67522 (14)	0.0262 (6)	
H10C	0.6768	0.7935	0.6772	0.031*	
H10D	0.7588	0.7055	0.6556	0.031*	
C11B	0.6503 (4)	0.6971 (2)	0.74369 (14)	0.0261 (6)	
H11C	0.7569	0.7153	0.7697	0.031*	
H11D	0.6497	0.6342	0.7424	0.031*	
C12B	0.4889 (4)	0.72799 (18)	0.77626 (14)	0.0215 (6)	
C13B	0.4411 (4)	0.68052 (18)	0.83786 (13)	0.0216 (6)	
H13B	0.4561	0.6184	0.8294	0.026*	
C14B	0.2412 (4)	0.6963 (2)	0.83999 (13)	0.0244 (6)	
H14C	0.1824	0.6446	0.8548	0.029*	
H14D	0.2194	0.7435	0.8698	0.029*	
C15B	0.1708 (4)	0.71938 (19)	0.77054 (13)	0.0235 (6)	
H15C	0.0718	0.6821	0.7558	0.028*	
H15D	0.1320	0.7794	0.7678	0.028*	
C16B	0.3282 (3)	0.70463 (18)	0.73089 (13)	0.0196 (5)	
H16B	0.3348	0.6417	0.7245	0.024*	
C17B	0.3284 (3)	0.74356 (18)	0.66389 (13)	0.0191 (5)	
H17B	0.3353	0.8068	0.6687	0.023*	
C18B	0.4960 (4)	0.8432 (2)	0.55865 (15)	0.0295 (7)	
H18D	0.5977	0.8651	0.5847	0.044*	
H18E	0.3885	0.8651	0.5755	0.044*	
H18F	0.5020	0.8616	0.5139	0.044*	
C19B	0.5012 (4)	0.82440 (19)	0.78917 (15)	0.0265 (6)	
H19D	0.6030	0.8362	0.8190	0.040*	
H19E	0.3947	0.8438	0.8081	0.040*	
H19F	0.5138	0.8545	0.7486	0.040*	
C20B	0.7436 (4)	0.6881 (2)	0.89911 (14)	0.0269 (6)	
H20D	0.7886	0.7311	0.8706	0.040*	
H20E	0.7672	0.6313	0.8825	0.040*	
H20F	0.8014	0.6944	0.9423	0.040*	
C21B	0.5456 (4)	0.6999 (2)	0.90218 (13)	0.0246 (6)	
H21B	0.5240	0.7607	0.9133	0.030*	
C22B	0.4755 (4)	0.6440 (2)	0.95550 (14)	0.0251 (6)	
H22C	0.3482	0.6543	0.9565	0.030*	
H22D	0.4913	0.5836	0.9439	0.030*	
C23B	0.5618 (4)	0.6585 (2)	1.02329 (14)	0.0287 (7)	
H23C	0.6844	0.6383	1.0249	0.034*	
H23D	0.5638	0.7202	1.0328	0.034*	
C24B	0.4650 (5)	0.6122 (2)	1.07447 (14)	0.0302 (7)	
H24C	0.4507	0.5518	1.0616	0.036*	
H24D	0.3465	0.6371	1.0755	0.036*	
C25B	0.5553 (5)	0.6157 (2)	1.14292 (16)	0.0365 (8)	
H25B	0.6779	0.5944	1.1411	0.044*	
C26B	0.5628 (6)	0.7051 (3)	1.16937 (17)	0.0534 (11)	
H26D	0.6242	0.7419	1.1405	0.080*	
H26E	0.6254	0.7050	1.2120	0.080*	
H26F	0.4436	0.7263	1.1728	0.080*	
C27B	0.4601 (7)	0.5578 (3)	1.18807 (19)	0.0580 (13)	
H27D	0.5212	0.5589	1.2310	0.087*	
H27E	0.4583	0.4995	1.1714	0.087*	
H27F	0.3398	0.5780	1.1907	0.087*	

### Atomic displacement parameters (Å^2^)

**Table:**

	*U*^11^	*U*^22^	*U*^33^	*U*^12^	*U*^13^	*U*^23^
Cl1A	0.0440 (4)	0.0481 (5)	0.0199 (3)	−0.0101 (4)	0.0024 (3)	0.0046 (3)
O1A	0.0234 (10)	0.0345 (12)	0.0354 (12)	−0.0065 (9)	0.0053 (9)	0.0060 (10)
C1A	0.0146 (11)	0.0246 (14)	0.0259 (13)	0.0015 (11)	0.0011 (9)	0.0000 (12)
C2A	0.0166 (12)	0.0205 (13)	0.0273 (14)	0.0028 (10)	0.0034 (10)	0.0006 (11)
C3A	0.0212 (12)	0.0173 (13)	0.0239 (13)	0.0019 (11)	0.0031 (10)	0.0034 (11)
C4A	0.0274 (14)	0.0250 (16)	0.0216 (13)	−0.0011 (12)	0.0035 (10)	0.0016 (11)
C5A	0.0319 (15)	0.0252 (16)	0.0200 (13)	−0.0048 (12)	0.0026 (11)	0.0028 (11)
C6A	0.0267 (14)	0.0394 (19)	0.0178 (13)	−0.0039 (14)	−0.0021 (10)	0.0076 (13)
C7A	0.0205 (13)	0.0388 (18)	0.0208 (13)	−0.0023 (13)	0.0002 (10)	0.0045 (13)
C8A	0.0176 (12)	0.0204 (14)	0.0200 (13)	−0.0008 (10)	0.0005 (10)	0.0026 (10)
C9A	0.0172 (11)	0.0225 (14)	0.0181 (12)	−0.0011 (11)	−0.0010 (9)	0.0020 (11)
C10A	0.0199 (13)	0.0332 (17)	0.0191 (13)	0.0020 (12)	−0.0021 (10)	0.0009 (12)
C11A	0.0181 (12)	0.0290 (16)	0.0209 (13)	0.0008 (11)	0.0016 (10)	0.0000 (11)
C12A	0.0182 (12)	0.0164 (13)	0.0207 (12)	0.0012 (10)	0.0001 (9)	0.0004 (10)
C13A	0.0224 (13)	0.0172 (13)	0.0181 (12)	0.0014 (10)	−0.0027 (10)	−0.0002 (10)
C14A	0.0213 (13)	0.0246 (15)	0.0228 (13)	−0.0010 (11)	−0.0026 (10)	−0.0009 (11)
C15A	0.0200 (12)	0.0246 (14)	0.0224 (12)	0.0012 (12)	−0.0019 (10)	0.0005 (11)
C16A	0.0209 (12)	0.0171 (13)	0.0168 (11)	0.0017 (10)	0.0010 (9)	0.0005 (10)
C17A	0.0153 (11)	0.0153 (12)	0.0217 (13)	0.0003 (9)	0.0014 (9)	0.0002 (10)
C18A	0.0323 (16)	0.0240 (16)	0.0268 (15)	0.0067 (12)	0.0016 (12)	0.0036 (12)
C19A	0.0296 (15)	0.0165 (14)	0.0274 (15)	0.0029 (11)	0.0031 (11)	−0.0011 (11)
C20A	0.0273 (14)	0.0244 (14)	0.0206 (12)	0.0035 (12)	0.0033 (11)	−0.0010 (11)
C21A	0.0240 (13)	0.0184 (14)	0.0228 (13)	−0.0009 (11)	0.0008 (10)	−0.0023 (11)
C22A	0.0234 (14)	0.0220 (15)	0.0226 (14)	0.0025 (11)	−0.0027 (11)	−0.0030 (11)
C23A	0.0302 (15)	0.0225 (15)	0.0199 (13)	0.0018 (12)	0.0025 (11)	−0.0001 (11)
C24A	0.0341 (16)	0.0190 (14)	0.0248 (15)	0.0034 (12)	−0.0001 (12)	0.0024 (11)
C25A	0.0387 (17)	0.0258 (15)	0.0199 (13)	−0.0024 (13)	0.0030 (12)	0.0006 (11)
C26A	0.058 (2)	0.0294 (18)	0.0263 (15)	0.0085 (17)	0.0001 (14)	0.0005 (14)
C27A	0.071 (3)	0.0285 (18)	0.0296 (18)	0.0049 (18)	0.0075 (17)	0.0050 (14)
Cl1B	0.0535 (5)	0.0531 (6)	0.0259 (4)	0.0118 (5)	0.0082 (3)	0.0052 (4)
O1B	0.0227 (10)	0.0377 (13)	0.0343 (12)	0.0052 (10)	−0.0006 (9)	0.0068 (10)
C1B	0.0143 (12)	0.0191 (14)	0.0290 (14)	0.0005 (10)	0.0009 (10)	−0.0004 (11)
C2B	0.0179 (12)	0.0193 (13)	0.0287 (14)	−0.0021 (11)	−0.0009 (10)	−0.0009 (11)
C3B	0.0205 (12)	0.0191 (14)	0.0269 (14)	−0.0008 (11)	0.0006 (10)	0.0012 (12)
C4B	0.0247 (14)	0.0267 (16)	0.0288 (15)	0.0016 (12)	0.0000 (11)	0.0023 (12)
C5B	0.0380 (17)	0.0318 (17)	0.0230 (14)	0.0086 (14)	0.0065 (12)	0.0055 (13)
C6B	0.0270 (15)	0.044 (2)	0.0341 (17)	0.0089 (14)	0.0107 (13)	0.0086 (15)
C7B	0.0196 (13)	0.0380 (18)	0.0296 (15)	0.0026 (13)	0.0036 (11)	0.0057 (14)
C8B	0.0164 (12)	0.0223 (14)	0.0278 (15)	0.0011 (11)	0.0018 (10)	0.0040 (12)
C9B	0.0155 (12)	0.0193 (14)	0.0299 (15)	−0.0012 (10)	0.0006 (10)	−0.0009 (12)
C10B	0.0127 (12)	0.0359 (18)	0.0300 (15)	−0.0024 (11)	0.0022 (10)	0.0025 (13)
C11B	0.0179 (12)	0.0279 (16)	0.0324 (15)	0.0025 (12)	0.0001 (11)	0.0005 (13)
C12B	0.0205 (13)	0.0193 (14)	0.0247 (14)	−0.0006 (10)	0.0022 (10)	−0.0001 (11)
C13B	0.0233 (13)	0.0170 (13)	0.0245 (13)	−0.0011 (11)	0.0014 (10)	−0.0039 (11)
C14B	0.0207 (13)	0.0258 (15)	0.0270 (14)	−0.0014 (12)	0.0025 (10)	−0.0014 (12)
C15B	0.0197 (13)	0.0233 (15)	0.0278 (14)	0.0007 (11)	0.0034 (10)	−0.0025 (11)
C16B	0.0163 (12)	0.0178 (13)	0.0249 (13)	−0.0003 (11)	0.0012 (9)	−0.0013 (11)
C17B	0.0145 (11)	0.0181 (13)	0.0246 (13)	0.0002 (10)	0.0002 (10)	−0.0016 (11)
C18B	0.0288 (15)	0.0231 (16)	0.0362 (17)	−0.0059 (12)	0.0003 (13)	0.0070 (13)
C19B	0.0264 (14)	0.0200 (14)	0.0323 (16)	−0.0036 (11)	−0.0033 (12)	−0.0015 (12)
C20B	0.0249 (14)	0.0268 (15)	0.0282 (14)	−0.0038 (12)	−0.0029 (11)	−0.0012 (12)
C21B	0.0251 (14)	0.0197 (14)	0.0283 (14)	0.0001 (12)	−0.0025 (11)	−0.0033 (12)
C22B	0.0302 (15)	0.0207 (15)	0.0241 (14)	−0.0014 (12)	−0.0003 (11)	−0.0015 (12)
C23B	0.0342 (17)	0.0252 (16)	0.0268 (15)	0.0019 (13)	0.0023 (12)	−0.0033 (12)
C24B	0.0440 (19)	0.0231 (16)	0.0232 (15)	−0.0032 (13)	−0.0005 (13)	−0.0012 (12)
C25B	0.044 (2)	0.0359 (19)	0.0290 (16)	−0.0013 (16)	0.0009 (14)	0.0014 (14)
C26B	0.087 (3)	0.048 (2)	0.0254 (17)	−0.032 (2)	0.0062 (18)	−0.0002 (17)
C27B	0.104 (4)	0.036 (2)	0.032 (2)	−0.020 (2)	−0.004 (2)	0.0072 (16)

### Geometric parameters (Å, º)

**Table:**

Cl1A—C5A	1.804 (3)	Cl1B—C5B	1.805 (3)
O1A—C2A	1.221 (3)	O1B—C2B	1.209 (3)
C1A—C2A	1.510 (4)	C1B—C2B	1.502 (4)
C1A—C17A	1.540 (4)	C1B—C17B	1.544 (4)
C1A—H1AA	0.9900	C1B—H1BA	0.9900
C1A—H1AB	0.9900	C1B—H1BB	0.9900
C2A—C3A	1.506 (4)	C2B—C3B	1.513 (4)
C3A—C4A	1.530 (4)	C3B—C4B	1.526 (4)
C3A—C8A	1.558 (4)	C3B—C8B	1.551 (4)
C3A—H3AA	1.0000	C3B—H3BA	1.0000
C4A—C5A	1.523 (4)	C4B—C5B	1.507 (4)
C4A—H4AA	0.9900	C4B—H4BA	0.9900
C4A—H4AB	0.9900	C4B—H4BB	0.9900
C5A—C6A	1.515 (4)	C5B—C6B	1.525 (5)
C5A—H5AA	1.0000	C5B—H5BA	1.0000
C6A—C7A	1.540 (4)	C6B—C7B	1.541 (4)
C6A—H6AA	0.9900	C6B—H6BA	0.9900
C6A—H6AB	0.9900	C6B—H6BB	0.9900
C7A—C8A	1.542 (4)	C7B—C8B	1.542 (4)
C7A—H7AA	0.9900	C7B—H7BA	0.9900
C7A—H7AB	0.9900	C7B—H7BB	0.9900
C8A—C18A	1.542 (4)	C8B—C18B	1.545 (4)
C8A—C9A	1.551 (4)	C8B—C9B	1.552 (4)
C9A—C10A	1.526 (4)	C9B—C10B	1.529 (4)
C9A—C17A	1.542 (4)	C9B—C17B	1.534 (4)
C9A—H9AA	1.0000	C9B—H9BA	1.0000
C10A—C11A	1.535 (4)	C10B—C11B	1.530 (4)
C10A—H10A	0.9900	C10B—H10C	0.9900
C10A—H10B	0.9900	C10B—H10D	0.9900
C11A—C12A	1.533 (4)	C11B—C12B	1.531 (4)
C11A—H11A	0.9900	C11B—H11C	0.9900
C11A—H11B	0.9900	C11B—H11D	0.9900
C12A—C19A	1.538 (4)	C12B—C19B	1.541 (4)
C12A—C16A	1.544 (4)	C12B—C16B	1.542 (4)
C12A—C13A	1.569 (4)	C12B—C13B	1.551 (4)
C13A—C21A	1.548 (4)	C13B—C21B	1.542 (4)
C13A—C14A	1.552 (4)	C13B—C14B	1.555 (4)
C13A—H13A	1.0000	C13B—H13B	1.0000
C14A—C15A	1.555 (4)	C14B—C15B	1.551 (4)
C14A—H14A	0.9900	C14B—H14C	0.9900
C14A—H14B	0.9900	C14B—H14D	0.9900
C15A—C16A	1.524 (4)	C15B—C16B	1.527 (4)
C15A—H15A	0.9900	C15B—H15C	0.9900
C15A—H15B	0.9900	C15B—H15D	0.9900
C16A—C17A	1.522 (4)	C16B—C17B	1.525 (4)
C16A—H16A	1.0000	C16B—H16B	1.0000
C17A—H17A	1.0000	C17B—H17B	1.0000
C18A—H18A	0.9800	C18B—H18D	0.9800
C18A—H18B	0.9800	C18B—H18E	0.9800
C18A—H18C	0.9800	C18B—H18F	0.9800
C19A—H19A	0.9800	C19B—H19D	0.9800
C19A—H19B	0.9800	C19B—H19E	0.9800
C19A—H19C	0.9800	C19B—H19F	0.9800
C20A—C21A	1.538 (4)	C20B—C21B	1.534 (4)
C20A—H20A	0.9800	C20B—H20D	0.9800
C20A—H20B	0.9800	C20B—H20E	0.9800
C20A—H20C	0.9800	C20B—H20F	0.9800
C21A—C22A	1.540 (4)	C21B—C22B	1.543 (4)
C21A—H21A	1.0000	C21B—H21B	1.0000
C22A—C23A	1.533 (4)	C22B—C23B	1.534 (4)
C22A—H22A	0.9900	C22B—H22C	0.9900
C22A—H22B	0.9900	C22B—H22D	0.9900
C23A—C24A	1.530 (4)	C23B—C24B	1.527 (4)
C23A—H23A	0.9900	C23B—H23C	0.9900
C23A—H23B	0.9900	C23B—H23D	0.9900
C24A—C25A	1.527 (4)	C24B—C25B	1.542 (5)
C24A—H24A	0.9900	C24B—H24C	0.9900
C24A—H24B	0.9900	C24B—H24D	0.9900
C25A—C26A	1.517 (5)	C25B—C26B	1.509 (6)
C25A—C27A	1.527 (5)	C25B—C27B	1.531 (5)
C25A—H25A	1.0000	C25B—H25B	1.0000
C26A—H26A	0.9800	C26B—H26D	0.9800
C26A—H26B	0.9800	C26B—H26E	0.9800
C26A—H26C	0.9800	C26B—H26F	0.9800
C27A—H27A	0.9800	C27B—H27D	0.9800
C27A—H27B	0.9800	C27B—H27E	0.9800
C27A—H27C	0.9800	C27B—H27F	0.9800
			
C2A—C1A—C17A	113.1 (2)	C2B—C1B—C17B	113.9 (2)
C2A—C1A—H1AA	109.0	C2B—C1B—H1BA	108.8
C17A—C1A—H1AA	109.0	C17B—C1B—H1BA	108.8
C2A—C1A—H1AB	109.0	C2B—C1B—H1BB	108.8
C17A—C1A—H1AB	109.0	C17B—C1B—H1BB	108.8
H1AA—C1A—H1AB	107.8	H1BA—C1B—H1BB	107.7
O1A—C2A—C3A	122.7 (3)	O1B—C2B—C1B	123.2 (3)
O1A—C2A—C1A	122.0 (3)	O1B—C2B—C3B	121.9 (3)
C3A—C2A—C1A	115.3 (2)	C1B—C2B—C3B	114.9 (2)
C2A—C3A—C4A	112.4 (2)	C2B—C3B—C4B	112.9 (2)
C2A—C3A—C8A	109.8 (2)	C2B—C3B—C8B	109.4 (2)
C4A—C3A—C8A	114.6 (2)	C4B—C3B—C8B	114.5 (2)
C2A—C3A—H3AA	106.5	C2B—C3B—H3BA	106.5
C4A—C3A—H3AA	106.5	C4B—C3B—H3BA	106.5
C8A—C3A—H3AA	106.5	C8B—C3B—H3BA	106.5
C5A—C4A—C3A	109.1 (2)	C5B—C4B—C3B	109.7 (2)
C5A—C4A—H4AA	109.9	C5B—C4B—H4BA	109.7
C3A—C4A—H4AA	109.9	C3B—C4B—H4BA	109.7
C5A—C4A—H4AB	109.9	C5B—C4B—H4BB	109.7
C3A—C4A—H4AB	109.9	C3B—C4B—H4BB	109.7
H4AA—C4A—H4AB	108.3	H4BA—C4B—H4BB	108.2
C6A—C5A—C4A	112.3 (3)	C4B—C5B—C6B	112.2 (3)
C6A—C5A—Cl1A	109.8 (2)	C4B—C5B—Cl1B	110.4 (2)
C4A—C5A—Cl1A	109.7 (2)	C6B—C5B—Cl1B	109.6 (2)
C6A—C5A—H5AA	108.3	C4B—C5B—H5BA	108.2
C4A—C5A—H5AA	108.3	C6B—C5B—H5BA	108.2
Cl1A—C5A—H5AA	108.3	Cl1B—C5B—H5BA	108.2
C5A—C6A—C7A	110.4 (2)	C5B—C6B—C7B	110.1 (3)
C5A—C6A—H6AA	109.6	C5B—C6B—H6BA	109.6
C7A—C6A—H6AA	109.6	C7B—C6B—H6BA	109.6
C5A—C6A—H6AB	109.6	C5B—C6B—H6BB	109.6
C7A—C6A—H6AB	109.6	C7B—C6B—H6BB	109.6
H6AA—C6A—H6AB	108.1	H6BA—C6B—H6BB	108.2
C6A—C7A—C8A	113.4 (2)	C6B—C7B—C8B	113.7 (2)
C6A—C7A—H7AA	108.9	C6B—C7B—H7BA	108.8
C8A—C7A—H7AA	108.9	C8B—C7B—H7BA	108.8
C6A—C7A—H7AB	108.9	C6B—C7B—H7BB	108.8
C8A—C7A—H7AB	108.9	C8B—C7B—H7BB	108.8
H7AA—C7A—H7AB	107.7	H7BA—C7B—H7BB	107.7
C18A—C8A—C7A	109.9 (2)	C7B—C8B—C18B	109.7 (3)
C18A—C8A—C9A	111.1 (2)	C7B—C8B—C3B	108.0 (2)
C7A—C8A—C9A	110.3 (2)	C18B—C8B—C3B	110.4 (2)
C18A—C8A—C3A	110.7 (2)	C7B—C8B—C9B	110.0 (2)
C7A—C8A—C3A	107.3 (2)	C18B—C8B—C9B	111.0 (3)
C9A—C8A—C3A	107.5 (2)	C3B—C8B—C9B	107.7 (2)
C10A—C9A—C17A	110.7 (2)	C10B—C9B—C17B	110.9 (2)
C10A—C9A—C8A	114.7 (2)	C10B—C9B—C8B	115.3 (2)
C17A—C9A—C8A	112.7 (2)	C17B—C9B—C8B	112.4 (2)
C10A—C9A—H9AA	106.0	C10B—C9B—H9BA	105.8
C17A—C9A—H9AA	106.0	C17B—C9B—H9BA	105.8
C8A—C9A—H9AA	106.0	C8B—C9B—H9BA	105.8
C9A—C10A—C11A	114.0 (2)	C9B—C10B—C11B	113.7 (2)
C9A—C10A—H10A	108.7	C9B—C10B—H10C	108.8
C11A—C10A—H10A	108.7	C11B—C10B—H10C	108.8
C9A—C10A—H10B	108.7	C9B—C10B—H10D	108.8
C11A—C10A—H10B	108.7	C11B—C10B—H10D	108.8
H10A—C10A—H10B	107.6	H10C—C10B—H10D	107.7
C12A—C11A—C10A	112.4 (2)	C10B—C11B—C12B	112.7 (2)
C12A—C11A—H11A	109.1	C10B—C11B—H11C	109.1
C10A—C11A—H11A	109.1	C12B—C11B—H11C	109.1
C12A—C11A—H11B	109.1	C10B—C11B—H11D	109.1
C10A—C11A—H11B	109.1	C12B—C11B—H11D	109.1
H11A—C11A—H11B	107.9	H11C—C11B—H11D	107.8
C11A—C12A—C19A	111.3 (2)	C11B—C12B—C19B	110.3 (2)
C11A—C12A—C16A	106.5 (2)	C11B—C12B—C16B	106.7 (2)
C19A—C12A—C16A	112.2 (2)	C19B—C12B—C16B	112.2 (2)
C11A—C12A—C13A	116.8 (2)	C11B—C12B—C13B	117.4 (2)
C19A—C12A—C13A	109.8 (2)	C19B—C12B—C13B	110.1 (2)
C16A—C12A—C13A	99.7 (2)	C16B—C12B—C13B	99.7 (2)
C21A—C13A—C14A	112.3 (2)	C21B—C13B—C12B	119.1 (2)
C21A—C13A—C12A	119.1 (2)	C21B—C13B—C14B	113.3 (2)
C14A—C13A—C12A	103.7 (2)	C12B—C13B—C14B	103.7 (2)
C21A—C13A—H13A	107.1	C21B—C13B—H13B	106.7
C14A—C13A—H13A	107.1	C12B—C13B—H13B	106.7
C12A—C13A—H13A	107.1	C14B—C13B—H13B	106.7
C13A—C14A—C15A	107.2 (2)	C15B—C14B—C13B	106.8 (2)
C13A—C14A—H14A	110.3	C15B—C14B—H14C	110.4
C15A—C14A—H14A	110.3	C13B—C14B—H14C	110.4
C13A—C14A—H14B	110.3	C15B—C14B—H14D	110.4
C15A—C14A—H14B	110.3	C13B—C14B—H14D	110.4
H14A—C14A—H14B	108.5	H14C—C14B—H14D	108.6
C16A—C15A—C14A	103.4 (2)	C16B—C15B—C14B	103.5 (2)
C16A—C15A—H15A	111.1	C16B—C15B—H15C	111.1
C14A—C15A—H15A	111.1	C14B—C15B—H15C	111.1
C16A—C15A—H15B	111.1	C16B—C15B—H15D	111.1
C14A—C15A—H15B	111.1	C14B—C15B—H15D	111.1
H15A—C15A—H15B	109.0	H15C—C15B—H15D	109.0
C17A—C16A—C15A	118.8 (2)	C17B—C16B—C15B	119.1 (2)
C17A—C16A—C12A	113.8 (2)	C17B—C16B—C12B	114.4 (2)
C15A—C16A—C12A	105.1 (2)	C15B—C16B—C12B	105.0 (2)
C17A—C16A—H16A	106.1	C17B—C16B—H16B	105.8
C15A—C16A—H16A	106.1	C15B—C16B—H16B	105.8
C12A—C16A—H16A	106.1	C12B—C16B—H16B	105.8
C16A—C17A—C1A	111.4 (2)	C16B—C17B—C9B	109.1 (2)
C16A—C17A—C9A	108.7 (2)	C16B—C17B—C1B	111.6 (2)
C1A—C17A—C9A	111.7 (2)	C9B—C17B—C1B	111.6 (2)
C16A—C17A—H17A	108.3	C16B—C17B—H17B	108.2
C1A—C17A—H17A	108.3	C9B—C17B—H17B	108.2
C9A—C17A—H17A	108.3	C1B—C17B—H17B	108.2
C8A—C18A—H18A	109.5	C8B—C18B—H18D	109.5
C8A—C18A—H18B	109.5	C8B—C18B—H18E	109.5
H18A—C18A—H18B	109.5	H18D—C18B—H18E	109.5
C8A—C18A—H18C	109.5	C8B—C18B—H18F	109.5
H18A—C18A—H18C	109.5	H18D—C18B—H18F	109.5
H18B—C18A—H18C	109.5	H18E—C18B—H18F	109.5
C12A—C19A—H19A	109.5	C12B—C19B—H19D	109.5
C12A—C19A—H19B	109.5	C12B—C19B—H19E	109.5
H19A—C19A—H19B	109.5	H19D—C19B—H19E	109.5
C12A—C19A—H19C	109.5	C12B—C19B—H19F	109.5
H19A—C19A—H19C	109.5	H19D—C19B—H19F	109.5
H19B—C19A—H19C	109.5	H19E—C19B—H19F	109.5
C21A—C20A—H20A	109.5	C21B—C20B—H20D	109.5
C21A—C20A—H20B	109.5	C21B—C20B—H20E	109.5
H20A—C20A—H20B	109.5	H20D—C20B—H20E	109.5
C21A—C20A—H20C	109.5	C21B—C20B—H20F	109.5
H20A—C20A—H20C	109.5	H20D—C20B—H20F	109.5
H20B—C20A—H20C	109.5	H20E—C20B—H20F	109.5
C20A—C21A—C22A	111.2 (2)	C20B—C21B—C13B	113.2 (2)
C20A—C21A—C13A	112.6 (2)	C20B—C21B—C22B	110.9 (2)
C22A—C21A—C13A	109.3 (2)	C13B—C21B—C22B	109.1 (2)
C20A—C21A—H21A	107.8	C20B—C21B—H21B	107.8
C22A—C21A—H21A	107.8	C13B—C21B—H21B	107.8
C13A—C21A—H21A	107.8	C22B—C21B—H21B	107.8
C23A—C22A—C21A	115.6 (2)	C23B—C22B—C21B	115.4 (3)
C23A—C22A—H22A	108.4	C23B—C22B—H22C	108.4
C21A—C22A—H22A	108.4	C21B—C22B—H22C	108.4
C23A—C22A—H22B	108.4	C23B—C22B—H22D	108.4
C21A—C22A—H22B	108.4	C21B—C22B—H22D	108.4
H22A—C22A—H22B	107.4	H22C—C22B—H22D	107.5
C24A—C23A—C22A	111.6 (2)	C24B—C23B—C22B	111.9 (3)
C24A—C23A—H23A	109.3	C24B—C23B—H23C	109.2
C22A—C23A—H23A	109.3	C22B—C23B—H23C	109.2
C24A—C23A—H23B	109.3	C24B—C23B—H23D	109.2
C22A—C23A—H23B	109.3	C22B—C23B—H23D	109.2
H23A—C23A—H23B	108.0	H23C—C23B—H23D	107.9
C25A—C24A—C23A	115.3 (3)	C23B—C24B—C25B	114.9 (3)
C25A—C24A—H24A	108.4	C23B—C24B—H24C	108.5
C23A—C24A—H24A	108.4	C25B—C24B—H24C	108.5
C25A—C24A—H24B	108.4	C23B—C24B—H24D	108.5
C23A—C24A—H24B	108.4	C25B—C24B—H24D	108.5
H24A—C24A—H24B	107.5	H24C—C24B—H24D	107.5
C26A—C25A—C24A	111.7 (3)	C26B—C25B—C27B	109.7 (3)
C26A—C25A—C27A	110.2 (3)	C26B—C25B—C24B	112.0 (3)
C24A—C25A—C27A	110.7 (3)	C27B—C25B—C24B	110.2 (3)
C26A—C25A—H25A	108.0	C26B—C25B—H25B	108.3
C24A—C25A—H25A	108.0	C27B—C25B—H25B	108.3
C27A—C25A—H25A	108.0	C24B—C25B—H25B	108.3
C25A—C26A—H26A	109.5	C25B—C26B—H26D	109.5
C25A—C26A—H26B	109.5	C25B—C26B—H26E	109.5
H26A—C26A—H26B	109.5	H26D—C26B—H26E	109.5
C25A—C26A—H26C	109.5	C25B—C26B—H26F	109.5
H26A—C26A—H26C	109.5	H26D—C26B—H26F	109.5
H26B—C26A—H26C	109.5	H26E—C26B—H26F	109.5
C25A—C27A—H27A	109.5	C25B—C27B—H27D	109.5
C25A—C27A—H27B	109.5	C25B—C27B—H27E	109.5
H27A—C27A—H27B	109.5	H27D—C27B—H27E	109.5
C25A—C27A—H27C	109.5	C25B—C27B—H27F	109.5
H27A—C27A—H27C	109.5	H27D—C27B—H27F	109.5
H27B—C27A—H27C	109.5	H27E—C27B—H27F	109.5
			
C17A—C1A—C2A—O1A	−131.1 (3)	C17B—C1B—C2B—O1B	−130.8 (3)
C17A—C1A—C2A—C3A	48.7 (3)	C17B—C1B—C2B—C3B	47.8 (3)
O1A—C2A—C3A—C4A	−5.1 (4)	O1B—C2B—C3B—C4B	−6.2 (4)
C1A—C2A—C3A—C4A	175.2 (2)	C1B—C2B—C3B—C4B	175.2 (2)
O1A—C2A—C3A—C8A	123.7 (3)	O1B—C2B—C3B—C8B	122.7 (3)
C1A—C2A—C3A—C8A	−56.0 (3)	C1B—C2B—C3B—C8B	−56.0 (3)
C2A—C3A—C4A—C5A	−177.3 (2)	C2B—C3B—C4B—C5B	−177.8 (3)
C8A—C3A—C4A—C5A	56.4 (3)	C8B—C3B—C4B—C5B	56.1 (3)
C3A—C4A—C5A—C6A	−56.4 (3)	C3B—C4B—C5B—C6B	−56.9 (4)
C3A—C4A—C5A—Cl1A	−178.8 (2)	C3B—C4B—C5B—Cl1B	−179.5 (2)
C4A—C5A—C6A—C7A	56.8 (4)	C4B—C5B—C6B—C7B	56.8 (4)
Cl1A—C5A—C6A—C7A	179.2 (2)	Cl1B—C5B—C6B—C7B	179.8 (2)
C5A—C6A—C7A—C8A	−56.2 (4)	C5B—C6B—C7B—C8B	−55.2 (4)
C6A—C7A—C8A—C18A	−67.0 (3)	C6B—C7B—C8B—C18B	−68.3 (4)
C6A—C7A—C8A—C9A	170.2 (3)	C6B—C7B—C8B—C3B	52.1 (4)
C6A—C7A—C8A—C3A	53.4 (3)	C6B—C7B—C8B—C9B	169.4 (3)
C2A—C3A—C8A—C18A	−62.2 (3)	C2B—C3B—C8B—C7B	179.1 (2)
C4A—C3A—C8A—C18A	65.4 (3)	C4B—C3B—C8B—C7B	−53.0 (3)
C2A—C3A—C8A—C7A	178.0 (2)	C2B—C3B—C8B—C18B	−60.9 (3)
C4A—C3A—C8A—C7A	−54.4 (3)	C4B—C3B—C8B—C18B	67.0 (3)
C2A—C3A—C8A—C9A	59.3 (3)	C2B—C3B—C8B—C9B	60.3 (3)
C4A—C3A—C8A—C9A	−173.1 (2)	C4B—C3B—C8B—C9B	−171.7 (2)
C18A—C8A—C9A—C10A	−66.0 (3)	C7B—C8B—C9B—C10B	53.7 (3)
C7A—C8A—C9A—C10A	56.0 (3)	C18B—C8B—C9B—C10B	−67.9 (3)
C3A—C8A—C9A—C10A	172.7 (2)	C3B—C8B—C9B—C10B	171.2 (2)
C18A—C8A—C9A—C17A	61.8 (3)	C7B—C8B—C9B—C17B	−177.9 (2)
C7A—C8A—C9A—C17A	−176.1 (2)	C18B—C8B—C9B—C17B	60.5 (3)
C3A—C8A—C9A—C17A	−59.4 (3)	C3B—C8B—C9B—C17B	−60.4 (3)
C17A—C9A—C10A—C11A	51.8 (3)	C17B—C9B—C10B—C11B	52.3 (3)
C8A—C9A—C10A—C11A	−179.3 (2)	C8B—C9B—C10B—C11B	−178.5 (3)
C9A—C10A—C11A—C12A	−53.4 (3)	C9B—C10B—C11B—C12B	−53.9 (4)
C10A—C11A—C12A—C19A	−68.2 (3)	C10B—C11B—C12B—C19B	−68.1 (3)
C10A—C11A—C12A—C16A	54.3 (3)	C10B—C11B—C12B—C16B	54.0 (3)
C10A—C11A—C12A—C13A	164.6 (2)	C10B—C11B—C12B—C13B	164.7 (2)
C11A—C12A—C13A—C21A	81.2 (3)	C11B—C12B—C13B—C21B	78.0 (3)
C19A—C12A—C13A—C21A	−46.7 (3)	C19B—C12B—C13B—C21B	−49.3 (3)
C16A—C12A—C13A—C21A	−164.6 (2)	C16B—C12B—C13B—C21B	−167.4 (2)
C11A—C12A—C13A—C14A	−153.2 (2)	C11B—C12B—C13B—C14B	−155.0 (2)
C19A—C12A—C13A—C14A	78.9 (3)	C19B—C12B—C13B—C14B	77.6 (3)
C16A—C12A—C13A—C14A	−39.0 (3)	C16B—C12B—C13B—C14B	−40.4 (3)
C21A—C13A—C14A—C15A	149.2 (2)	C21B—C13B—C14B—C15B	151.8 (2)
C12A—C13A—C14A—C15A	19.3 (3)	C12B—C13B—C14B—C15B	21.3 (3)
C13A—C14A—C15A—C16A	8.7 (3)	C13B—C14B—C15B—C16B	6.9 (3)
C14A—C15A—C16A—C17A	−163.1 (2)	C14B—C15B—C16B—C17B	−162.9 (2)
C14A—C15A—C16A—C12A	−34.3 (3)	C14B—C15B—C16B—C12B	−33.2 (3)
C11A—C12A—C16A—C17A	−60.5 (3)	C11B—C12B—C16B—C17B	−58.9 (3)
C19A—C12A—C16A—C17A	61.5 (3)	C19B—C12B—C16B—C17B	62.1 (3)
C13A—C12A—C16A—C17A	177.6 (2)	C13B—C12B—C16B—C17B	178.6 (2)
C11A—C12A—C16A—C15A	167.8 (2)	C11B—C12B—C16B—C15B	168.7 (2)
C19A—C12A—C16A—C15A	−70.2 (3)	C19B—C12B—C16B—C15B	−70.4 (3)
C13A—C12A—C16A—C15A	46.0 (3)	C13B—C12B—C16B—C15B	46.1 (3)
C15A—C16A—C17A—C1A	−50.7 (3)	C15B—C16B—C17B—C9B	−175.1 (2)
C12A—C16A—C17A—C1A	−175.3 (2)	C12B—C16B—C17B—C9B	59.6 (3)
C15A—C16A—C17A—C9A	−174.3 (2)	C15B—C16B—C17B—C1B	−51.4 (3)
C12A—C16A—C17A—C9A	61.1 (3)	C12B—C16B—C17B—C1B	−176.7 (2)
C2A—C1A—C17A—C16A	−167.2 (2)	C10B—C9B—C17B—C16B	−53.4 (3)
C2A—C1A—C17A—C9A	−45.4 (3)	C8B—C9B—C17B—C16B	175.9 (2)
C10A—C9A—C17A—C16A	−53.8 (3)	C10B—C9B—C17B—C1B	−177.1 (2)
C8A—C9A—C17A—C16A	176.2 (2)	C8B—C9B—C17B—C1B	52.2 (3)
C10A—C9A—C17A—C1A	−177.3 (2)	C2B—C1B—C17B—C16B	−166.8 (2)
C8A—C9A—C17A—C1A	52.8 (3)	C2B—C1B—C17B—C9B	−44.5 (3)
C14A—C13A—C21A—C20A	−178.5 (2)	C12B—C13B—C21B—C20B	−55.1 (4)
C12A—C13A—C21A—C20A	−57.1 (3)	C14B—C13B—C21B—C20B	−177.4 (3)
C14A—C13A—C21A—C22A	57.3 (3)	C12B—C13B—C21B—C22B	−179.1 (2)
C12A—C13A—C21A—C22A	178.7 (2)	C14B—C13B—C21B—C22B	58.6 (3)
C20A—C21A—C22A—C23A	56.4 (3)	C20B—C21B—C22B—C23B	57.0 (3)
C13A—C21A—C22A—C23A	−178.7 (2)	C13B—C21B—C22B—C23B	−177.7 (2)
C21A—C22A—C23A—C24A	172.0 (2)	C21B—C22B—C23B—C24B	170.3 (3)
C22A—C23A—C24A—C25A	176.8 (3)	C22B—C23B—C24B—C25B	173.6 (3)
C23A—C24A—C25A—C26A	66.8 (4)	C23B—C24B—C25B—C26B	65.1 (4)
C23A—C24A—C25A—C27A	−169.9 (3)	C23B—C24B—C25B—C27B	−172.6 (3)

### Hydrogen-bond geometry (Å, º)

**Table:**

*D*—H···*A*	*D*—H	H···*A*	*D*···*A*	*D*—H···*A*
C7*A*—H7*AA*···O1*A*^i^	0.99	2.37	3.349 (4)	168
C7*B*—H7*BA*···O1*B*^i^	0.99	2.47	3.265 (4)	137
C23*A*—H23*B*···O1*B*^ii^	0.99	2.51	3.414 (4)	152
C23*B*—H23*D*···O1*A*^iii^	0.99	2.50	3.453 (4)	161

Symmetry codes: (i) *x*+1, *y*, *z*; (ii) −*x*+1, *y*−1/2, −*z*+1; (iii) −*x*+1, *y*+1/2, −*z*+2.
